# Effects of functional characteristics of prickly ash leaves on yield following increased supply of nitrogen fertilizers

**DOI:** 10.3389/fpls.2026.1761126

**Published:** 2026-02-10

**Authors:** Junlan Liu, Guoqing Sun, Qin Huang, Haodan Zhang, Ailin Tian, Linyu Liu, Yun Ren, Qiang Li, Zexiong Chen

**Affiliations:** Chongqing Key Laboratory for Germplasm Innovation for Special Aromatic Spice Plants, College of Smart Agriculture/Institute of Special Plants, Chongqing University of Arts and Sciences, Chongqing, China

**Keywords:** contributing characteristics, leaf functional, nitrogen fertilizer, prickly ash, yield

## Abstract

This study investigated the effects of nitrogen fertilizer on leaf morphology, photosynthetic pigments, photosynthetic characteristics, and yield of prickly ash (*Zanthoxylum* L.) and clarified the differences in the contribution of leaf morphology, photosynthetic pigments, and photosynthetic characteristics to this yield of prickly ash. Using Jiuyeqing as the experimental material, three nitrogen fertilizer levels of N0, N120, and N240 kg ha^−1^ were established. The results showed that nitrogen application improved the morphological and structural traits of prickly ash leaves, enhanced the contents of photosynthetic pigments and photosynthetic performance, significantly increased the accumulation of dry matter and nitrogen in plants, and notably elevated the ear number, 100-grain weight, single-ear weight, and yield. Correlation analysis found Leaf area, chlorophyll content, and net photosynthetic rate were highly positively correlated with yield. Leaf area contributed the most to yield, followed by the net photosynthetic rate, whereas chlorophyll content contributed the least. The results of path analysis showed that leaf area (0.422), chlorophyll content (0.237), and net photosynthetic rate (-0.098) exerted relatively small direct effects on the yield of prickly ash. Specifically, these three leaf traits, namely leaf area, chlorophyll content, and net photosynthetic rate, indirectly regulated the yield via nitrogen accumulation, with the corresponding indirect path coefficients being 1.656, 1.601, and 1.645, respectively. Therefore, nitrogen accumulation in prickly ash increased significantly after nitrogen application, which increased the photosynthetic leaf area, chlorophyll content, photosynthetic rate, and yield.

## Graphical Abstract

**Figure:**
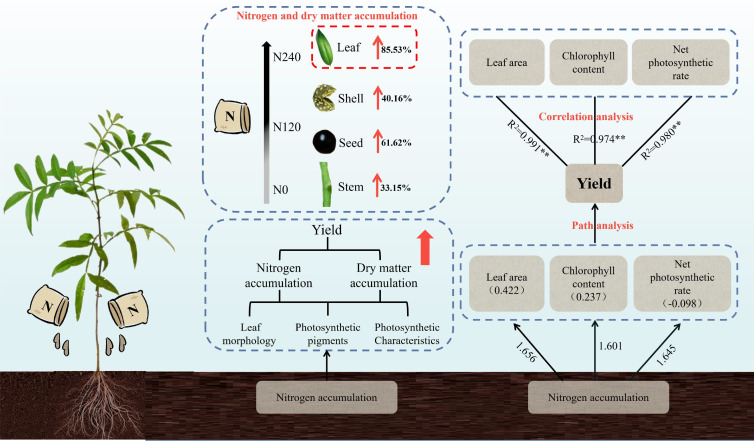

## 1 Introduction

Prickly ash, a small perennial tree belonging to the genus *Zanthoxylum* (Rutaceae), has fruits and leaves rich in volatile organic compounds, such as terpenoids and alcohols, and has a unique aromatic and pungent taste (Satria et al., 2019). It has become a multipurpose economic plant used as a source of spices, seasonings, and in traditional Chinese medicine. It is widely cultivated worldwide (Bao et al., 2023; Ma et al., 2022). China is the largest producer of prickly ash, with the largest planting area and yield. Red and green prickly ashes are consumed. Red prickly ash, which originated in cold northern areas such as the Taihang Mountains, Qinba Mountains, and Gansu Province, has small, light-colored leaves. In contrast green prickly ash originated in warm southwestern areas, including Chongqing, the western Sichuan Plateau, and the Yunnan Guizhou Plateau (Cao et al., 2019). Green prickly ash has large and thick leaves with a well-developed root system that is resistant to drought and poor soil conditions (Dong et al., 2024). It has become an important carrier of rural revitalization and ecological protection in the mountainous areas of southwest China (Deng et al., 2025).

Nitrogen is not only a component of macromolecular organic matter, such as nucleic acids, amino acids, and proteins in plants, but also the main component of active substances, such as chlorophyll, hormones, and vitamins in plants (Jia et al., 2017; Melzer, 2021). Nitrogen supply significantly affects the nitrogen content and distribution in plants. Appropriate nitrogen supply promotes plant growth and development and dry matter accumulation, increases photosynthetic pigment content in leaves, improves photosynthetic efficiency, and finally achieves high yield and high quality (Zheng et al., 2025). Rong et al. (2020) showed that nitrogen application could significantly increase the biomass of roots, stems, and leaves of Fujian cypress. Wang et al. (2020) found that nitrogen application could significantly increase the relative chlorophyll content of the hybrid hazelnut, regulate the chlorophyll fluorescence characteristic parameters (F0, Fv and Fm), and improve the vertical diameter, transverse diameter, lateral diameter, single fruit weight, kernel weight and kernel rate ratio of hazelnut, so as to promote the high yield and high quality of hazelnut. In contrast, excessive or insufficient nitrogen supply inhibits growth and development, affects normal photosynthesis in plants, and ultimately leads to crop yield reduction (Meng et al., 2022, Wang (2025)) found that nitrogen deficiency can reduce cotton plant height and stem diameter. Excessive nitrogen fertilizer can lead to excessive growth of cotton, and it will also reduce the distribution ratio of ‘ sink ‘ organs in the late growth stage, ultimately reducing the yield and quality of cotton. Therefore, precise regulation for nitrogen supply is the core technology of high-yield and high-quality cultivation of crops, which is crucial for improving crop production efficiency. The above studies confirmed the core role of nitrogen in regulating morphological structure, material accumulation, photosynthetic efficiency, yield and quality, but the correlation between leaf functional traits and yield under nitrogen regulation was not clear.

Leaves are the most important photosynthetic organs in plants, and their functional traits directly determine crop yield and quality (Li et al., 2024). Photosynthesis is the most basic life activity for maintaining the normal growth and development of plants, and chlorophyll composed of nitrogen plays a key role in carbon assimilation during plant photosynthesis (Rana et al., 2022). Mo (2023) pointed out that nitrogen application can increase the leaf length, leaf width, leaf area, specific leaf weight, and leaf dry weight of alfalfa and improve the net photosynthetic rate, transpiration rate, and stomatal conductance of leaves, thereby improving the photosynthetic area and photosynthetic rate and increasing the yield of prickly ash. This is consistent with the results of Sun et al. (2022) for alfalfa. Therefore, nitrogen is involved in chlorophyll synthesis and regulates the photosynthetic parameters of leaves. It also affects leaf morphogenesis, which in turn affects the yield formation and quality improvement of crops. The above studies found that nitrogen regulation of leaf functional traits is the core way to affect yield, but the existing conclusions are mostly based on herbaceous plants, and the response of woody plants to prickly ash still needs to be explored.

Existing research has mainly focused on the effects of nitrogen fertilizer on leaf development, chlorophyll content, photosynthetic parameters, and the yield of prickly ash. However, the interaction between leaf morphology (leaf area) and physiological traits (chlorophyll content and photosynthetic parameters) under regulation and their specific contributions to yield formation remain unclear. Therefore, in this study, the main cultivar,’ Jiuyeqing ‘ prickly ash, in southwest China was used as the experimental material to explore the effects of nitrogen fertilizer on leaf morphology, chlorophyll content, photosynthetic parameters, and yield. The direct and indirect effects of various traits and yield formation were determined by path analysis. The purpose of this study was to reveal the contribution characteristics of leaf functional traits to yield under nitrogen regulation, to provide a theoretical basis and technical support for the efficient utilization of nitrogen nutrition in prickly ash.

## 2 Materials and methods

### 2.1 Overview of the test site

The experiment was conducted at the prickly ash test base of the Chongqing University of Arts and Sciences, Yongchuan District, Chongqing City (29°17’N, 105°84’E), in 2023 and 2024. The experimental area has a subtropical monsoon humid climate, with an annual average temperature of 17.7°C, annual average precipitation of 1015 mm, annual average sunshine of 1218.7 h, and an annual average frost-free period of 317 days. The soil was purple, and the nutrient content of the upper soil layer (0 to 30 cm) was as follows: quick-acting nitrogen 36.51 mg kg^-1^, quick-acting phosphorus 105.33 mg kg^-1^, quick-acting potassium 265.72 mg kg^-1^, total nitrogen 1.13 g kg^-1^, total phosphorus 15.72 g kg^-1^, total potassium 1.83 g kg^-1^, organic matter 16.21 g kg^-1^; soil pH was 7.01.

### 2.2 Experimental design

**Figure 1:**
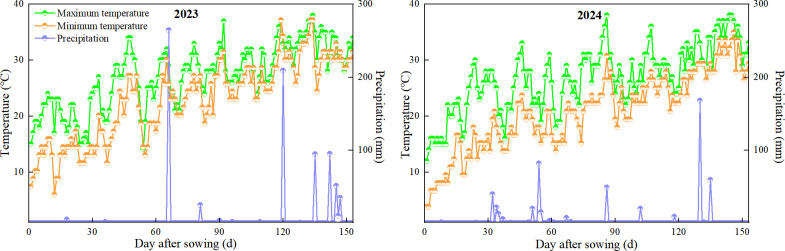
Meteorological factors during the Chinese prickly ash test.

The experiment was designed as a single-factor randomized block design, and the test variety was ‘Jiuyeqing’ (*Zanthoxylum* L.). Three nitrogen fertilizer levels were applied as treatments: N0 (0 kg ha^−1^), N120 (120 kg ha^−1^), and N240 (240 kg ha^−1^). Each treatment was repeated thrice for nine plots, and each plot contained 40 prickly ash plants. The prickly ash garden was seven years old and had reached its peak production period with a row spacing of 3 m × 3 m and a density of 1300 plants ha^-1^. Application furrows were spaced 0.8 m apart from those in adjacent plots. Additionally, plastic films should be vertically buried between furrows to prevent lateral fertilizer seepage. The fertilization method involved digging trenches along the drip lines on both sides of the tree trunk, applying the fertilizer, mixing the fertilizer evenly with the soil, and refilling the trenches with a hoe. The amount of nitrogen fertilizer was determined according to the experimental design, and the amounts of phosphorus and potassium fertilizers were used according to local high-yield cultivation The amount of nitrogen fertilizer (urea) was based on the experimental design, while the amount of super-phosphate was 180 kg ha^-1^, and the amount of potassium chloride was 200 kg ha^-1^. The application period and amounts of nitrogen, phosphorus, and potassium fertilizers in each period are shown in (**Table 1**). Diseases, insects, and weeds were controlled according to local high-yield cultivation practices. The meteorological variables during the experiment are shown in (**Figure 1**).

**Table 1:** Application period and amount of nitrogen, phosphorus and potassium fertilizer for each treatment.

Treatment		ABF (kg·ha^−1^)	OF (kg·ha^−1^)	PFF (kg·ha^−1^)	SFF (kg·ha^−1^)	Total (kg·ha^−1^)
N	N0	0	0	0	0	0
	N120	28	32	28	32	120
	N240	55	65	55	65	240
P		40	60	40	40	180
K		40	60	55	45	200

ABF, Autumn base fertilizer; OF, Overwinter fertilizer; PFF, Promote flower fat; SFF, Strong fruit fat.

### 2.3 Indicator measurement

#### 2.3.1 Leaf morphological

Prickly ash trees were divided into three parts after fruit set, and each branch was divided into three sections: upper, middle, and lower. Thirty undamaged leaves without, pests or disease were selected. Leaf length, width, fresh weight, and dry weight were measured, and the leaf area and leaf shape index were calculated. The test results for the three periods were averaged.

$\text{LA }(\text{c}{\text{m}}^{2})=\text{Leaf length }\left(\text{cm}\right)\times \text{Leaf width }\left(\text{cm}\right)\times 0.75$


$$\text{LI}=\frac{\text{Leaf length}}{\text{Leaf width}}$$

#### 2.3.2 Determination of photosynthetic pigment content

The healthy leaves without damage and pests were selected from the upper, middle and lower sections of each branch, and the pigment content was determined by ethanol extraction method. The specific operation is as follows: 0.1 g prickly ash seedling leaves were weighed, cut into pieces and completely immersed in 95% ethanol and diluted to 10 mL. The leaves were soaked in dark at room temperature for 48 h until the leaves were completely decolorized and whitened. The absorbance of the extract was measured at 663 nm, 645 nm and 470 nm with 95% ethanol as the blank control, and each treatment was repeated three times. The contents of chlorophyll a, chlorophyll b, total chlorophyll and carotenoid were calculated according to Arnon formula (Xie, 2013).

#### 2.3.3 Determination of photosynthetic parameters

Measurements were conducted on prickly ash between 09:00 and 11:00 on clear days in June using a Li-6400 Portable Photosynthesis System. A red-blue light source leaf chamber was adopted with an open gas-exchange path. The leaf chamber parameters were set uniformly as follows: Light intensity of 1600 µmol m^-2^ s^-1^, chamber temperature of 25°C, CO_2_ concentration of 420 µmol mol^-1^, relative humidity of 60%, and flow rate of 500 µmol s^-1^. For each treatment, three plots were randomly chosen, and the upper, middle, and lower leaves of each plant were measured for net photosynthetic rate (*Pn*), stomatal conductance (*Gs*), transpiration rate (*Tr*), and intercellular CO_2_ concentration (*Ci*). The three leaf measurements from the same plot were averaged to obtain one plot-level value, giving three (n = 3) replicates per treatment. The plot means were used in the analysis of variance.

#### 2.3.4 Dry matter accumulation and distribution

Prickly ash was harvested by pruning the branches, and all branches, leaves, and fruits were collected. Only the main stem was retained, and changes in the growth of the main stem were neglected. After harvesting, the green prickly ash plants were divided into stems, leaves, and fruits (drying, the samples were separated into peels and seeds). The plants were heated at 105°C for 30 min and then transferred to an oven at 80°C until they reached a constant weight. Dry matter accumulation and distribution were calculated by weighing and then crushing through a 60-mesh sieve for the later determination of nitrogen content.

#### 2.3.5 Nitrogen accumulation and distribution

During the fruit-setting period of prickly ash, three healthy and consistently growing prickly ash plant samples were randomly selected from each plot. The stems, leaves, peels, and seeds were collected. The stems, leaves, peels, and seeds were killed at 105°C for 30 min and dried at 80°C to a constant quality. After drying, the powder was crushed into powder by grinding prototype, and the nitrogen content of each organ was determined using the Kjeldahl method, and the nitrogen accumulation was calculated (Gerhardt, 2015).


Nitrogen accumulation (mg/plant)=dry weight (mg/plant)×nitrogen content (%)

#### 2.3.6 Yield and component

All plants, except the side rows and sampling plants, were harvested during the harvest period of prickly ash, and 5 plants were retained in each plot for testing. The number of rows per ear, number of grains per row, number of grains per ear, and the 100-grain weight of the prickly ash were measured. The yield and grain weight were converted according to the national grain storage standard water content (13%), which was the actual yield.


$$\text{Yield}(\text{kg}/\text{acre})=\frac{\text{fresh weight (kg)}}{{\text{actual area of plot (m}}^{2})}\times 666.67\times 0.13$$

$$\text{Yield}(\text{t}/\text{h}{\text{a}}^{-1})=\frac{\text{Yield}(\text{kg}/\text{acre})}{66.667}$$

### 2.4 Data and analysis

Excel 2019 and Origin 2023 were used to collate and plot data. SPSS software (version 19.0) was used for the analysis of variance and one-way ANOVA. Path analysis was performed using DPS 7.05 software.

## 3 Results

### 3.1 Effect of nitrogen fertilizer on leaf morphology of prickly ash

Nitrogen application significantly increased leaf length, width, shape index, area, and fresh weight (**Table 2**). Compared with N0, the leaf length of N120 and N240 in 2023 increased by 24.6% and 36.3%, leaf width increased by 16.0% and 26.3%, the leaf shape index increased by 7.4% and 7.9%, the leaf area increased by 34.6% and 57.1%, and the leaf fresh weight increased by 21.5% and 39.8%, respectively; In 2024, these variables increased by 20.1% and 30.8%, 17.3% and 23.2%, 2.4% and 6.1%, 32.9% and 54.4%, 18.2% and 34.2%, respectively, The results showed that nitrogen fertilizer application could significantly promote the elongation and widening of prickly ash leaves, thereby increasing the leaf shape index and leaf area.

**Table 2:** Effects of different nitrogen fertilizer treatments on leaf phenotypic characteristics of prickly ash leaves.

Years	N levels	Leaf length (cm)	Leaf width (cm)	Leaf shape index	Leaf area (cm^2^)	Fresh leaf weight (g)
2023	N0	5.983 ± 0.140 c	1.913 ± 0.047 c	3.130 ± 0.062 b	9.083 ± 0.099 c	0.241 ± 0.004 c
	N120	7.453 ± 0.122 b	2.220 ± 0.046 b	3.357 ± 0.064 a	12.227 ± 0.127 b	0.293 ± 0.006 b
	N240	8.157 ± 0.833 a	2.417 ± 0.055 a	3.377 ± 0.047 a	14.270 ± 0.120 a	0.337 ± 0.005 a
2024	N0	6.290 ± 0.080 c	1.967 ± 0.045 c	3.197 ± 0.058 b	9.353 ± 0.063 c	0.257 ± 0.005 c
	N120	7.557 ± 0.093 b	2.307 ± 0.025 b	3.277 ± 0.045 b	12.433 ± 0.087 b	0.304 ± 0.006 b
	N240	8.227 ± 0.080 a	2.423 ± 0.031 a	3.393 ± 0.015 a	14.443 ± 0.075 a	0.352 ± 0.004 a
Years		*	NS	NS	**	**
N levels		**	**	**	**	**
Years ×N levels		NS	NS	NS	NS	NS

Different lowercase letters in the same column indicated significant differences (*p*<0.05), ** indicated extremely significant differences at a significant level of 1% (*p*<0.01), * indicated significant differences at a significant level of 5% (*p*<0.05), and “NS” indicated no significant difference.

### 3.2 Effects of nitrogen fertilizer on leaf pigment content and photosynthesis

**Figure 2:**
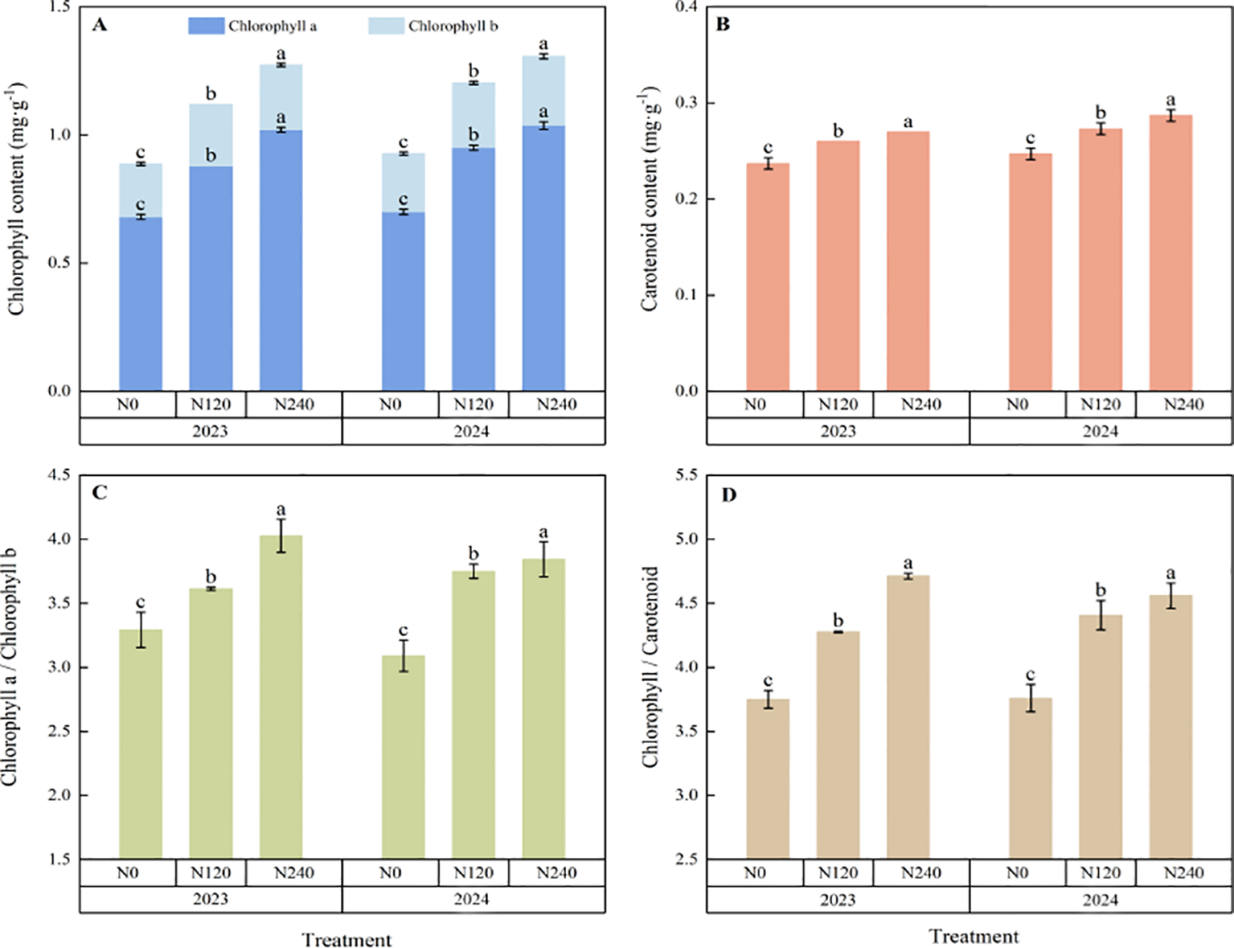
Effects of different nitrogen fertilization rates on physiological characteristics of prickly ash leaves. **(A)**: Effects of different nitrogen application rates on total chlorophyll, chlorophyll a, chlorophyll b of prickly ash leaves; **(B)**: Effects of different nitrogen application rates on carotenoid content in prickly ash leaves; **(C)**: Effects of different nitrogen application rates on chlorophyll a / chlorophyll b; **(D)**: Effects of different nitrogen application rates on total chlorophyll / carotenoids. The error bar represents the standard deviation ( SD ). Different lowercase letters ( a, b, c ) indicate significant differences between groups.

The chlorophyll *a*, chlorophyll *b*, total chlorophyll, carotenoid, chlorophyll *a/b*, and chlorophyll/carotenoid contents of the prickly ash leaves were significantly increased by nitrogen application (**Figure 2**). Compared with N0, chlorophyll *a* of N120 and N240 in 2023 increased by 29.4% and 50.0%, chlorophyll *b* increased by 15.9% and 22.2%, total chlorophyll increased by 26.3% and 43.5%, carotenoids increased by 9.7% and 13.9%, chlorophyll *a/b* increased by 9.7% and 22.3%, chlorophyll/carotenoids increased by 14.1% and 25.7%, respectively; in 2024, these variables increased by 35.7% and 48.1%, 11.5% and 18.9%, 29.8% and 41.0%, 10.5% and 16.2%, 21.4% and 24.4%, 17.2% and 21.3%.

**Figure 3:**
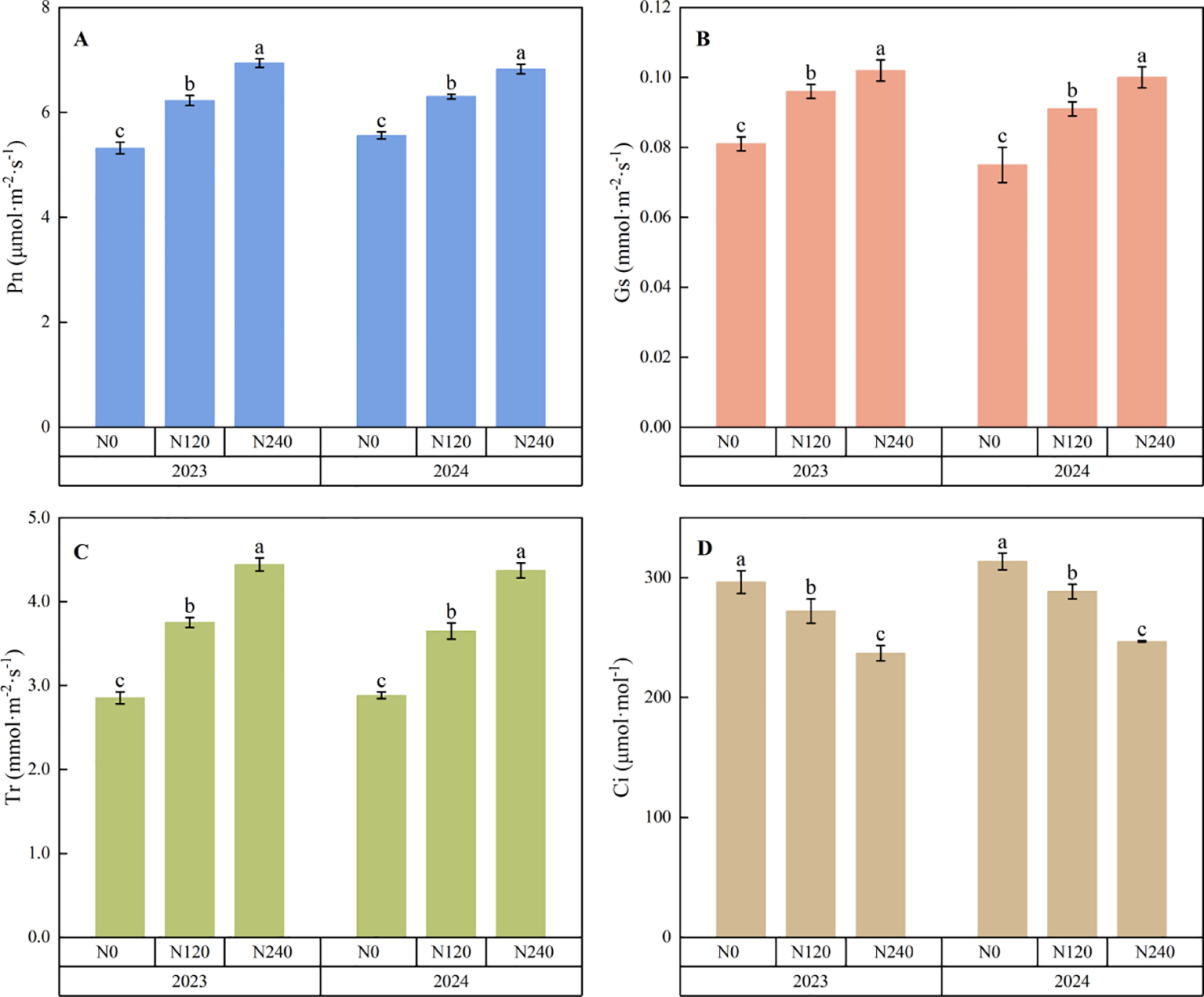
Photosynthetic gas exchange parameters of prickly ash leaves under different nitrogen fertilizer treatments. **(A)**: Effects of different nitrogen application rates on net photosynthetic rate; **(B)**: Effects of different nitrogen application rates on stomatal conductance; **(C)**: Effects of different nitrogen application rates on transpiration rate; **(D)**: Effects of different nitrogen application rates on intercellular CO2 concentration. The error bar represents the standard deviation ( SD ). Different lowercase letters ( a, b, c ) indicate significant differences between groups.

Nitrogen application significantly affected the photosynthetic characteristics of the prickly ash leaves. (**Figure 3**). Compared with N0, the net photosynthetic rates of N120 and N240 in 2023 increased by 17.0% and 30.5%, respectively, stomatal conductance increased by 17.6% and 25.0%, respectively, and transpiration rate increased by 275.3% and 344.3%, respectively. In 2024, these variables increased by 13.4%, 22.8%, 21.2%, 32.3%, 265.0%, and 337.8%, respectively, and the intracellular CO_2_ concentration decreased significantly. Compared with N0, the intracellular CO_2_ concentrations of N120 and N240 decreased by 8.2% and 20.0%, respectively, in 2023. In 2024, these variables were reduced by 8.0% and 21.3%, respectively. The results showed that the net photosynthetic rate, stomatal conductance, and transpiration rate of prickly ash increased significantly after nitrogen application, and the intercellular CO_2_ concentration decreased significantly.

### 3.3 Effects of nitrogen fertilizer on nitrogen and dry matter accumulation

**Figure 4:**
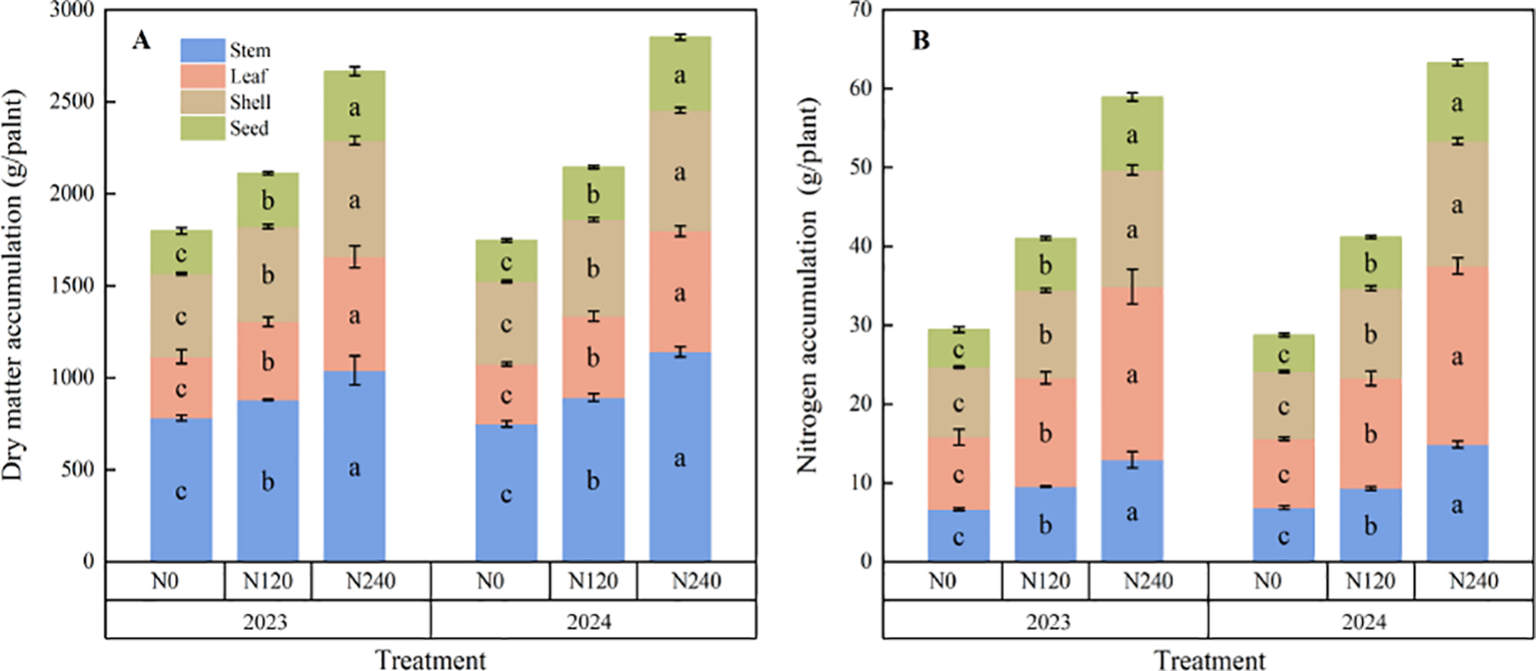
Dry matter accumulation and nitrogen accumulation in each organ under different treatments. **(A)**: Effects of different nitrogen application rates on dry matter accumulation in different organs of prickly ash; **(B)**: Effects of different nitrogen application rates on nitrogen accumulation in different organs of prickly ash. The error bar represents the standard deviation ( SD ). Different lowercase letters ( a, b, c ) indicate significant differences between groups.

After nitrogen application, nitrogen and dry matter accumulation in the stems, leaves, pericarp, seeds, and individual plants of prickly ash significantly increased (**Figure 4**). Compared with N0, the stem nitrogen accumulation of N120 and N240 increased by 43.5% and 94.4%, respectively, in 2023; leaf nitrogen accumulation increased by 50.5% and 139.6%, respectively; pericarp nitrogen accumulation increased by 25.1% and 66.8%, respectively; seed nitrogen accumulation increased by 38.8% and 94.5%, respectively; nitrogen accumulation per plant increased by 39.3% and 100.2%, respectively; and in 2024, they increased by 35.7% and 116.5%, 59.5% and 159.1%, 34.2% and 86.1%, 40.3% and 114.1%, 43.2% and 120.1%, respectively. Compared with N0, stem dry matter accumulation of N120 and N240 increased by 12.5% and 33.2%, respectively, in 2023; leaf dry matter accumulation increased by 27.6% and 85.5%, respectively; pericarp dry matter accumulation increased by 14.8% and 40.2%, respectively; seed dry matter accumulation increased by 24.9% and 61.6%, respectively; and the dry matter accumulation per plant increased by 17.5% and 48.3%, respectively. In 2024, these values increased by 19.1% and 52.3%, 36.3% and 102.2%, 17.1% and 46.3%, 26.3% and 77.1%, 63.2% and 22.7%, respectively. Thus, nitrogen application significantly increased nitrogen and dry matter accumulation in various organs of prickly ash plants, with the highest increase in nitrogen and dry matter accumulation in the leaves.

### 3.4 Effects of nitrogen fertilizer on yield and its components

The yield, grain number per spike, 100-grain weight, and single-spike weight of the prickly ash were significantly increased by nitrogen application (**Table 3**). Compared with N0, yields of N120 and N240 in 2023 increased by 49.6% and 89.3%, respectively; grain number per spike increased by 9.9% and 19.4%, respectively; 100-grain weight increased by 12.4% and 33.1%, respectively; and single-spike weight increased by 20.5% and 57.7%, respectively. In 2024, they will increase by 43.2% and 83.1%, 7.6% and 17.3%, 14.8% and 37.7%, 16.3% and 50.7%, The results showed that nitrogen application could effectively improve the yield and its composition of prickly ash, and the increase of 100-grain weight was significantly higher than that of grain number per spike.

**Table 3:** Effects of different nitrogen fertilizer treatments on the yield and composition of prickly ash.

Years	N levels	Yield (t·ha^−1^)	grain number per spike	100-grain weight (g)	Spike weight (g)
2023	N0	6.70 ± 0.15 c	46.51 ± 0.53 c	10.82 ± 0.17 c	5.41 ± 0.14 c
	N120	10.03 ± 0.26 b	51.09 ± 0.46 b	12.16 ± 0.22 b	6.52 ± 0.18 b
	N240	12.69 ± 0.27 a	55.54 ± 0.95 a	14.40 ± 0.12 a	8.53 ± 0.18 a
2024	N0	7.17 ± 0.20 c	48.65 ± 0.23 c	10.30 ± 0.17 c	5.85 ± 0.11 c
	N120	10.27 ± 0.40 b	52.34 ± 0.14 b	11.82 ± 0.12 b	6.81 ± 0.13 b
	N240	13.13 ± 0.32 a	57.08 ± 0.18 a	14.19 ± 0.20 a	8.82 ± 0.12 a
Years		NS	**	**	**
N levels		**	**	**	**
Years × N levels		NS	NS	NS	NS

Different lowercase letters in the same column indicated significant differences (*p*<0.05), ** indicated extremely significant differences at a significant level of 1% (*p*<0.01), * indicated significant differences at a significant level of 5% (*p*<0.05), and “NS” indicated no objection.

### 3.5 Correlation between leaf functional traits and yield

**Figure 5:**
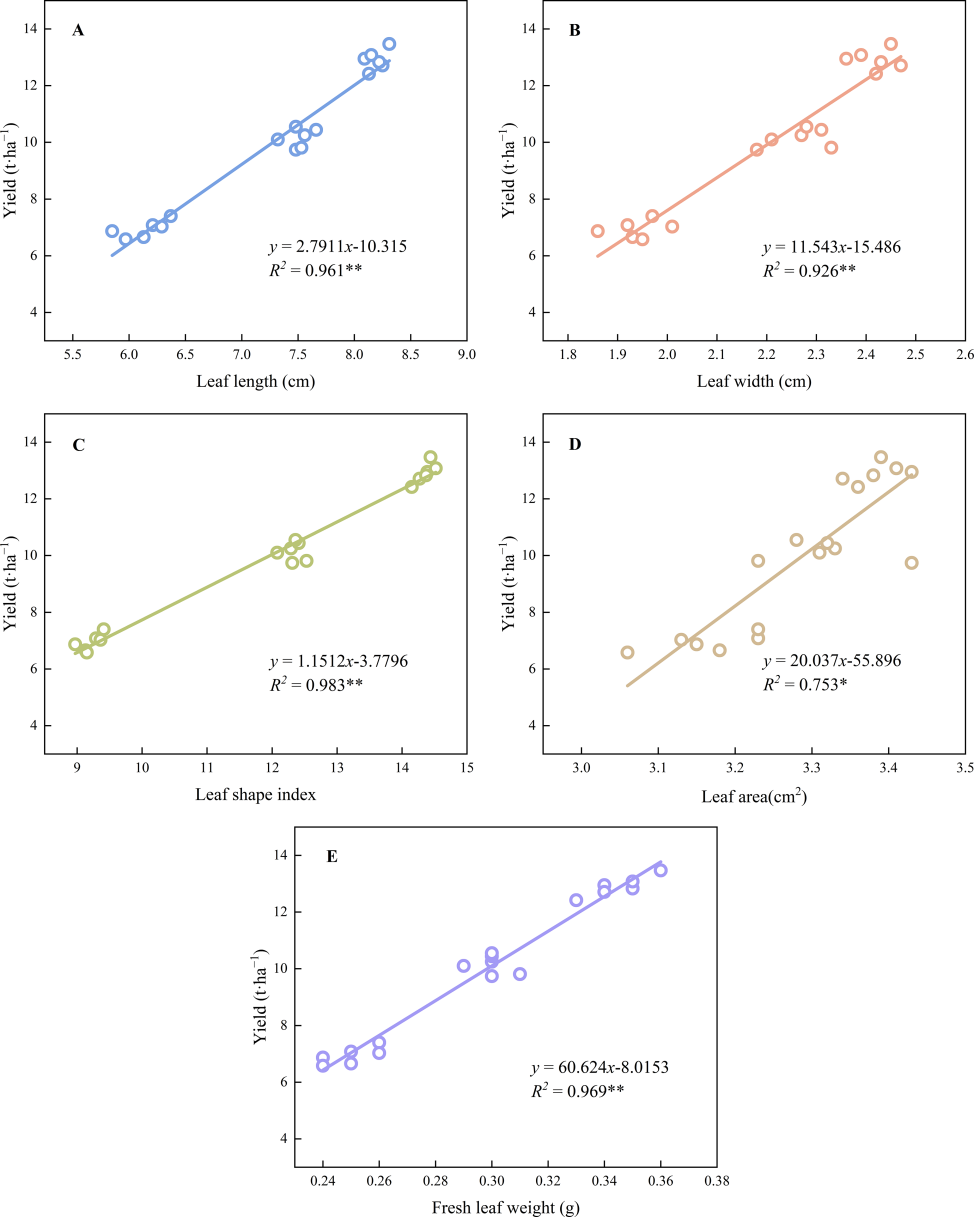
Relationship between photosynthetic pigment and yield. ** Indicated extremely significant differences at a significant level of 1% (*p*<0.01), * indicated significant differences at a significant level of 5% (*p*<0.05). The data points in the figure are the observed values of six experiments in 2023 and 2024, which are repeated three times a year for two consecutive years. **(A)**: Relationship between leaf length and yield. **(B)**: Relationship between leaf width and yield. **(C)**: Relationship between leaf shape index and yield. **(D)**: Relationship between leaf area and yield. **(E)**: The relationship between leaf fresh weight and yield. ** Indicated extremely significant differences at a significant level of 1% (p<0.01), * indicated significant differences at a significant level of 5% (p<0.05). The data points in the figure are the observed values of six experiments in 2023 and 2024, which are repeated three times a year for two consecutive years.

**Figure 6:**
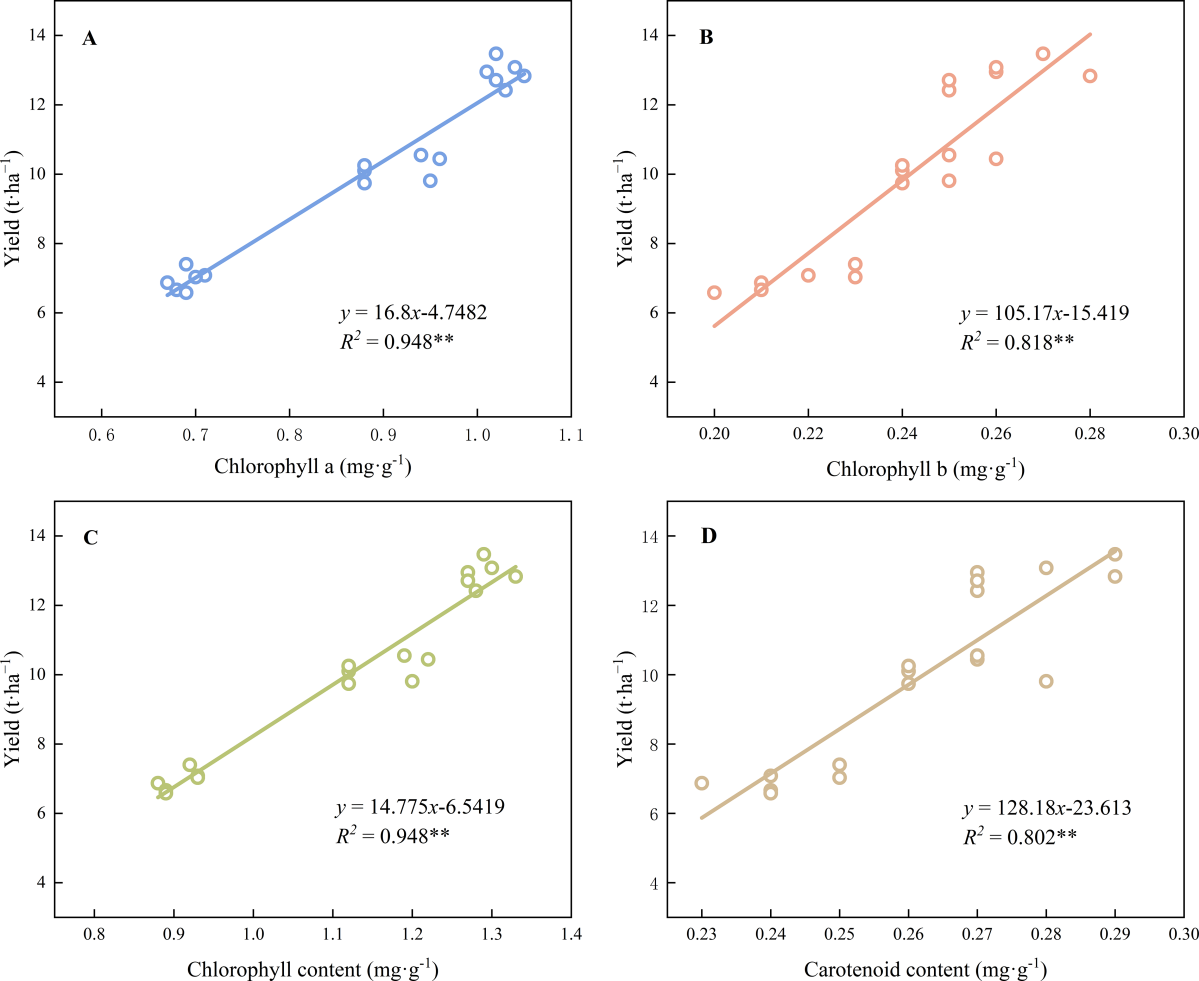
The relationship between leaf morphology and yield. ** Indicated extremely significant differences at a significant level of 1% (*p*<0.01), * indicated significant differences at a significant level of 5% (*p*<0.05). The data points in the figure are the observed values of six experiments in 2023 and 2024, which are repeated three times a year for two consecutive years. **(A)**: Relationship between chlorophyll a and yield. **(B)**: Relationship between chlorophyll b and yield. **(C)**: Relationship between total chlorophyll and yield. **(D)**: Relationship between carotenoids and yield. ** Indicated extremely significant differences at a significant level of 1% (p<0.01), * indicated significant differences at a significant level of 5% (p<0.05). The data points in the figure are the observed values of six experiments in 2023 and 2024, which are repeated three times a year for two consecutive years.

**Figure 7:**
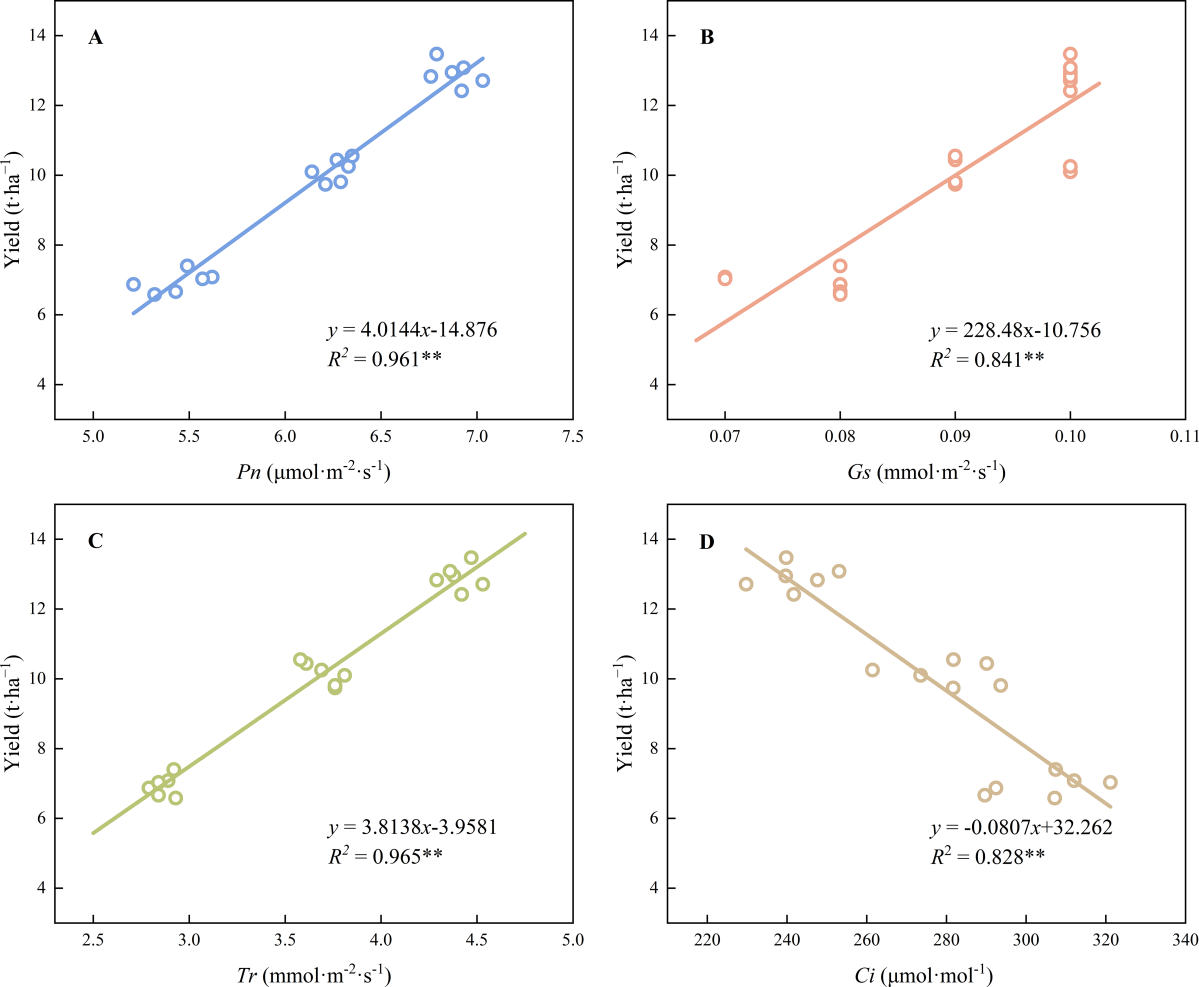
The relationship between photosynthetic parameters and yield. ** Indicated extremely significant differences at a significant level of 1% (*p*<0.01), * indicated significant differences at a significant level of 5% (*p*<0.05). The data points in the figure are the observed values of six experiments in 2023 and 2024, which are repeated three times a year for two consecutive years. **(A)**: Relationship between net photosynthetic rate and yield. **(B)**: Relationship between stomatal conductance and yield. **(C)**: Relationship between transpiration rate and yield. **(D)**: Relationship between intercellular CO2 concentration and yield. ** Indicated extremely significant differences at a significant level of 1% (p<0.01), * indicated significant differences at a significant level of 5% (p<0.05). The data points in the figure are the observed values of six experiments in 2023 and 2024, which are repeated three times a year for two consecutive years.

The contents of chlorophyll *a* (*R^2^* = 0.948 *p*< 0.01) and *b* (*R^2^* = 0.818 *p*< 0.01), total chlorophyll (*R^2^* = 0.948 *p*< 0.01), and carotenoids (*R^2^* = 0.802 *p*< 0.01) showed highly significant positive correlations with yield (**Figure 5**). Leaf length (*R^2^* = 0.961 *p*< 0.01), leaf width (*R^2^* = 0.926 *p*< 0.01), leaf shape index (*R^2^* = 0.983 *p*< 0.01), leaf area (*R^2^* = 0.753 *p*< 0.05), and leaf fresh weight (*R^2^* = 0.969 *p*< 0.01) were significantly and positively correlated with average yield (**Figure 6**). The net photosynthetic rate (*R^2^* = 0.961 *p*< 0.01), stomatal conductance (*R^2^* = 0.841 *p*< 0.01), and transpiration rate (*R^2^* = 0.965 *p*< 0.01) were significantly and positively correlated with yield, whereas intercellular CO_2_ concentration (*R^2^* = 0.828 *p*< 0.01) was significantly negatively correlated with yield (**Figure 7**), The results showed that leaf functional traits (morphological characteristics, photosynthetic pigments, photosynthetic characteristics) were significantly positively correlated with yield, whereas intercellular CO_2_ concentration was significantly negatively correlated with yield.

**Figure 8:**
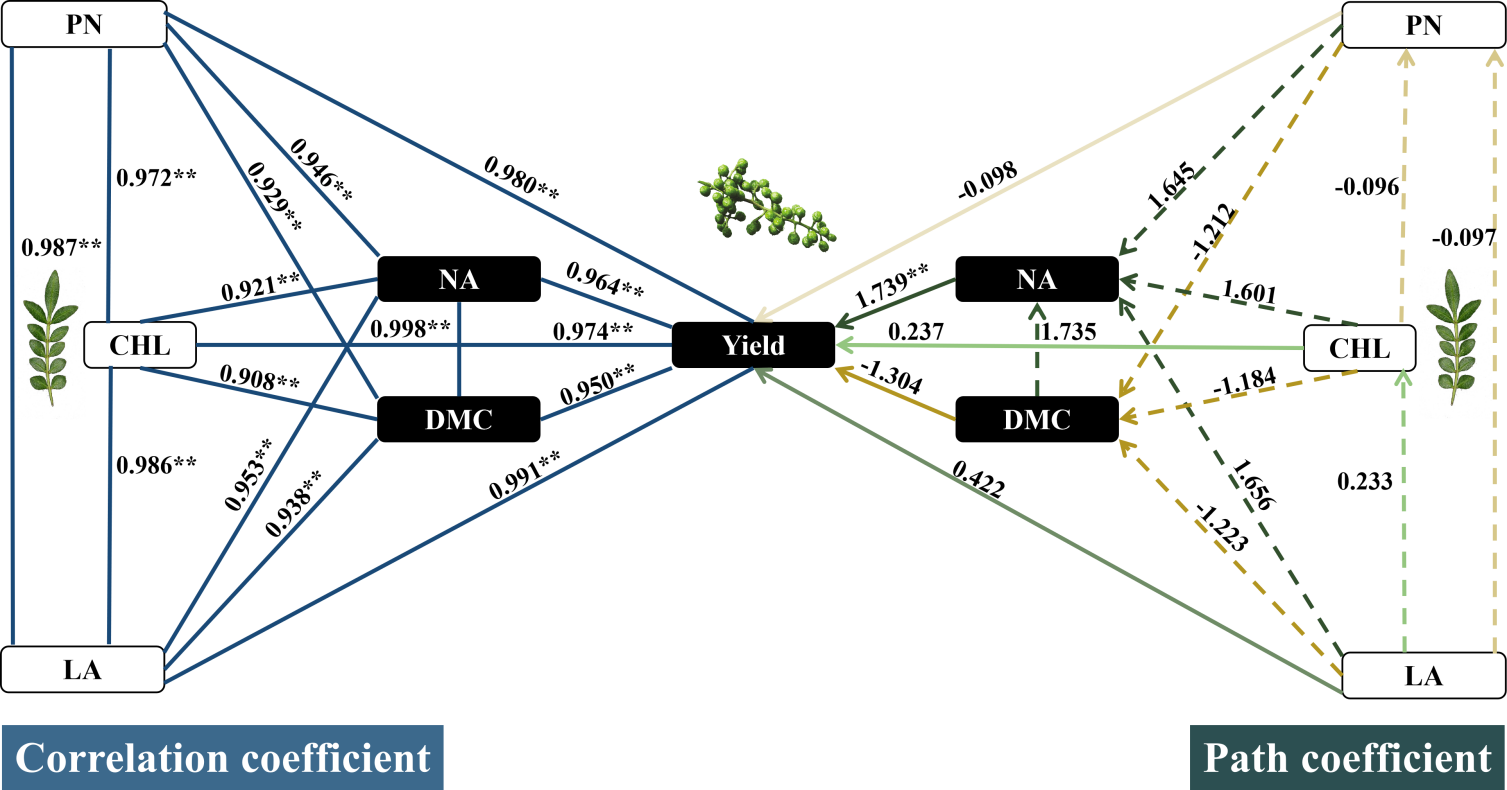
Path analysis and correlation analysis of the effect of leaf growth performance on yield of prickly ash. “—” The correlation between net photosynthetic rate, chlorophyll, leaf area, nitrogen accumulation, dry matter accumulation and yield was expresse0d; “” Indicates the direct path coefficient; “” Represents the indirect path coefficient. ** Indicated extremely significant differences at a significant level of 1% (*p*<0.01), * indicated significant differences at a significant level of 5% (*p*<0.05). DMC, dry matter accumulation; CHL, chlorophyll; PN, net photosynthetic rate; LA, leaf area; NA, nitrogen accumulation.

Leaf area, chlorophyll content, and net photosynthetic rate significantly affected the prickly ash yield (**Figure 8**). Leaf area (*R^2^* = 0.991 *p*< 0.01), net photosynthetic rate (*R^2^* = 0.980 *p*< 0.01), chlorophyll content (*R^2^* = 0.974 *p*< 0.01), nitrogen accumulation (*R^2^* = 0.964 *p*< 0.01), and dry matter accumulation (*R^2^* = 0.950 *p*< 0.01) were significantly and positively correlated with the prickly ash yield. Path analysis showed that leaf area (0.422) had the greatest direct effect on the yield of prickly ash, and net photosynthetic rate (-0.097), chlorophyll content (0.233), dry matter accumulation (-1.223), and nitrogen accumulation (1.656) indirectly affected the yield of prickly ash. The second largest determinant was chlorophyll content (0.237), and the yield of prickly ash was affected by the indirect effects of net photosynthetic rate (-0.096), leaf area (0.416), dry matter accumulation (-1.184), and nitrogen accumulation (1.601). The direct effect of the net photosynthetic rate (-0.098) on the yield of prickly ash was negative, but its correlation coefficient was positive because the indirect effects of chlorophyll (0.230) and nitrogen accumulation (1.645) had a larger positive effect on the yield of prickly ash. The results showed that the net photosynthetic rate, chlorophyll content and leaf area of prickly ash significantly affected the yield of prickly ash. The direct effect of leaf area was the largest, followed by chlorophyll content, and the net photosynthetic rate was the smallest. At the same time, the indirect effect of nitrogen accumulation on leaf functional traits affecting yield was the largest.

## 4 Discussion

### 4.1 Nitrogen-driven enhancement of photosynthetic capacity and source strength

Leaves are the main organs in plants that use solar energy for energy conversion, and their morphological structure directly determines the growth and development of plants. Nitrogen is the nutrient element with the highest demand for plant growth. Nitrogen application can lay the foundation for improving photosynthetic capacity by regulating the morphological structure of leaves (Regolo et al., 2024; Zhang et al., 2020). The results of this experiment showed that the leaf length, leaf width, leaf area, leaf shape index, and leaf fresh weight of prickly ash were significantly increased after nitrogen application, and that nitrogen could provide material support for plant cell division and volume expansion. Therefore, nitrogen application can effectively promote the expansion of plant leaves, which is consistent with previous research results on Sugarcane (Welutung et al., 2024), *Oryza sativa* (Lubis et al., 2024), Maize (Ajiboye and Oroka, 2025), and other plants. After nitrogen application, the leaf shape index of the prickly ash leaves increased significantly, and the increase in leaf length was greater than that of leaf width, which was inconsistent with the results of Li et al. (2021) on quinoa. As a woody plant, prickly ash has a higher degree of leaf lignification than quinoa. After nitrogen application, the leaves tend to expand along the main vein direction (longitudinal) rather than laterally, which is caused by the different growth and development of woody and cereal plants (Yu, 2021).

Nitrogen is involved in the synthesis of chlorophyll in leaves, which is a crucial indicator of leaf photosynthetic capacity (Liu et al., 2024). After nitrogen application, there were significant increases in chlorophyll a, chlorophyll b, total chlorophyll, carotenoid, and the ratios of chlorophyll a/b and total chlorophyll/carotenoid in the leaves of the prickly ash plants. This is because nitrogen is a vital component of chlorophyll synthesis (Ahmed, 2017). By enhancing chlorophyll synthesis, nitrogen promotes the synthesis of photosynthetic pigments and photosynthetic carbon assimilation, thereby improving plant photosynthetic activity and light-energy utilization. This finding is consistent with that of Sabbagh et al. (2015), who reported similar results for *Trachyspermum ammi*. After nitrogen application, the chlorophyll a/b ratio of prickly ash increased significantly, which was inconsistent with the results of Zhao et al. (2022) on *Styrax obassia*. As a shade-tolerant plant, *Styrax obassia* adapts to low-light environments by decreasing the ratio of chlorophyll a to chlorophyll b, whereas prickly ash, a heliophilic plant, adapts to high-light environments by increasing this ratio (Pang et al., 2024). It was further found that the total chlorophyll/carotenoid content increased significantly after nitrogen application, indicating that the increase of chlorophyll content was significantly higher than that of carotenoid content, because nitrogen was the direct raw material for chlorophyll synthesis, and the total chlorophyll content increased significantly after nitrogen application. As an indirect effect of regulating carotenoid synthesis, nitrogen has little direct effect; therefore, the increase in carotenoid content after nitrogen application was small (Rodríguez et al., 2009; Ortiz et al., 2009).

Nitrogen regulates CO_2_ and water exchange in plant leaves through stomatal openings (Zheng et al., 2020; Wada and Kong, 2018). The results of this experiment showed that the net photosynthetic rate, transpiration rate, and stomatal conductance of the prickly ash leaves increased significantly after nitrogen application, whereas the intercellular CO_2_ concentration decreased significantly, consistent with the results of Zhang et al. (2024) for sorghum. Nitrogen application accelerates the consumption of intercellular CO_2_, promotes an increase in stomatal conductance to supplement CO_2_, leads to an increase in transpiration rate, and finally increases the net photosynthetic rate (Li et al., 2013). However, Hao et al. (2015) found that under the treatment of high nitrogen N315 kg ha^-1^, the net photosynthetic rate, transpiration rate, and stomatal conductance showed a downward trend, and the intercellular CO_2_ concentration increased. Excessive nitrogen destroys the reaction center of photosystem II, reduces the utilization efficiency of photosynthetic nitrogen, limits stomatal conductance, thus inhibiting photosynthetic efficiency, and photosynthesis decreases significantly (Liu, 2008). The highest nitrogen application rate in this experiment was N240, but it did not reach this trend. The nitrogen application rate set in this experiment was still within the suitable nitrogen range for prickly ash, and the concentration of excessive nitrogen in prickly ash had not yet reached the inhibition threshold of excessive nitrogen.

### 4.2 Modulation of nitrogen partitioning and sink development under varied N supply

Nitrogen supply affects the regulation of crop nitrogen distribution and organ development and provides a material basis for yield formation (Ye et al., 2022). The results of this experiment showed that the nitrogen and dry matter accumulation of prickly ash plants significantly increased after nitrogen application. Further analysis showed that the nitrogen and dry matter accumulation of leaves in various organs of prickly ash increased the most after nitrogen application, which was consistent with the research results of Tao et al. (2025) on prickly ash, indicating that leaves are the main organs for photosynthesis in plants. After nitrogen application, nitrogen transport and distribution tend to be in leaves to meet the needs of photosynthesis, thereby improving the photosynthetic rate and carbon fixation capacity of leaves. The efficient synthesis of photosynthetic products provides sufficient material supply for grain development, thereby achieving yield growth (Li and Yu, 2019). After nitrogen application, single panicle weight, 100-grain weight, grain number per panicle, and total yield were significantly increased. After nitrogen application, the single spike weight, 100-grain weight, grain number per spike, and total yield were significantly increased. Nitrogen application promoted the improvement of crop yield and its components. By optimizing the efficiency of nitrogen uptake and translocation in plants, nitrogen application enhances the allocation and accumulation of nitrogen in fruits, which provides sufficient nutritional support for the increase in grain number per spike, 100-grain weight, and single spike weight. It also strengthened the translocation of photosynthetic products from various organs to the fruits, promoted the coordinated optimization of various yield components, and ultimately achieved a significant increase in the total yield. This result was consistent with the findings of Xing et al. (2025) on wheat. Correlation analysis revealed that nitrogen accumulation (*R^2^* = 0.964 *p*< 0.01) and dry matter accumulation (*R^2^* = 0.950 *p*< 0.01) were significantly positively correlated with yield, which was consistent with previous research results on maize (Yang et al., 2022), *Camellia oleifera* (Liu et al., 2022), and wheat (Li et al., 2020). Nitrogen is the main component of plant proteins and nucleotides (Makino et al., 2003). Nitrogen application increases the content of nitrogen-containing compounds in plants, affects the physiological activity and metabolic level of plants, accelerates the growth and development of various organs, improves the structure of the library, and ultimately promotes a significant increase in dry matter accumulation.

### 4.3 Integrated analysis of yield formation and resource use efficiency

Leaf morphology, pigment content, and photosynthetic characteristics of leaves are the core elements for measuring the functional traits, which significantly affect dry matter accumulation and plant yield (Fu et al., 2023). This experiment found that the leaf area (*R^2^* = 0.991 *p*< 0.01), total chlorophyll content (*R^2^* = 0.974 *p*< 0.01), and net photosynthetic rate (*R^2^* = 0.980 *p*< 0.01) of prickly ash were significantly and positively correlated with yield, consistent with the results of Zheng et al. (2024), Showing that the functional traits of leaves significantly affect the formation of crop yield. Leaf area reflects the efficiency of light energy capture, total chlorophyll content determines the efficiency of light energy utilization, and the net photosynthetic rate directly reflects photosynthetic efficiency. The three factors synergistically regulate the photosynthetic capacity of plant leaves and guarantee dry matter accumulation and yield formation (Lusk et al., 2019; Fang et al., 2025). Further analysis showed that the leaf area of prickly ash contributed the most to the grain yield, followed by the net photosynthetic rate, and the total chlorophyll content contributed the least. Xiong et al. (2022) found in the study of maize that chlorophyll content contributed the most to yield, followed by leaf area, and finally the results of net photosynthetic rate were inconsistent. Maize is a C4 plant with high photosynthetic efficiency that is sensitive to changes in chlorophyll. Prickly ash is a C3 plant with low photosynthetic efficiency per unit leaf, and photosynthesis is more dependent on the increase in leaf area. Therefore, leaf area has become an important leaf functional trait affects the yield of prickly ash (Li and Yu, 2019). In addition, through path analysis, it was further found that the direct effects of leaf area (0.422), chlorophyll content (0.237) and net photosynthetic rate (-0.098) on the yield of prickly ash were low or even negative, mainly through the indirect effects of nitrogen accumulation (1.656, 1.601 and 1.645) to regulate the yield of prickly ash, indicating that the effect of nitrogen application on the yield of prickly ash was not directly produced by optimizing the functional traits of leaves, but by increasing the nitrogen accumulation of prickly ash after nitrogen application as an indirect carrier to increase the photosynthetic leaf area, increase the chlorophyll content, increase the net photosynthetic rate, and finally increase the yield of prickly ash. This is consistent with the results of Hu et al. (2025) on rice.

## 5 Conclusion

Nitrogen application significantly optimizes the morphological structure characteristics (leaf length, leaf width, leaf shape index, leaf area, and leaf fresh weight), photosynthetic pigments (chlorophyll *a*, chlorophyll *b*, total chlorophyll, chlorophyll *a/b*, carotenoid, and total chlorophyll/carotenoid), and photosynthetic characteristics (net photosynthetic rate, transpiration rate, stomatal conductance, and intercellular CO_2_ concentration) of prickly ash leaves, effectively promoting a significant increase in dry matter and nitrogen accumulation of prickly ash, thereby significantly improving its grain number per spike, 100-grain weight, single spike weight, and yield. Correlation analysis showed that the yield of prickly ash was significantly and positively correlated with leaf area, chlorophyll content and net photosynthetic rate. Leaf area contributed the most to the increase in prickly ash yield, followed by net photosynthetic rate, whereas chlorophyll content contributed the least, which was significantly different from that of C4 plants. Therefore, the yield-increasing pathway of C3 plants should focus on increasing the leaf area. Path analysis verified the regulatory effect of nitrogen on the yield of prickly ash. The direct effects of leaf area, chlorophyll content, and net photosynthetic rate on the yield of prickly ash were low, and the yield was mainly regulated by the indirect effect of nitrogen accumulation. It was further confirmed that the nitrogen accumulation of plants was increased by increasing nitrogen fertilizer, and the photosynthetic traits of leaves (expanding leaf area, increasing chlorophyll content, and increasing photosynthetic rate) were optimized to finally achieve the regulation path of increasing yield.

## Data Availability

The original contributions presented in the study are included in the article/supplementary material. Further inquiries can be directed to the corresponding authors.
